# A positive feedback loop between TAZ and miR-942-3p modulates proliferation, angiogenesis, epithelial-mesenchymal transition process, glycometabolism and ROS homeostasis in human bladder cancer

**DOI:** 10.1186/s13046-021-01846-5

**Published:** 2021-01-26

**Authors:** Feifan Wang, Mengjing Fan, Xuejian Zhou, Yanlan Yu, Yueshu Cai, Hongshen Wu, Yan Zhang, Jiaxin Liu, Shihan Huang, Ning He, Zhenghui Hu, Guoqing Ding, Xiaodong Jin

**Affiliations:** 1 Department of Urology, The First Affiliated Hospital, Zhejiang University School of Medicine, Hangzhou Zhejiang, 310003 P.R. China; 2 Department of Pathology, Sir Run Run Shaw Hospital, Zhejiang University School of Medicine, Hangzhou Zhejiang, 310016 P.R. China; 3 Department of Urology, Sir Run Run Shaw Hospital, Zhejiang University School of Medicine, Hangzhou Zhejiang, 310016 P. R. China

**Keywords:** Bladder cancer, TAZ, miR-942-3p, Progression, Angiogenesis, EMT, Glycolysis, Reactive oxygen species

## Abstract

**Background:**

Transcriptional coactivator with PDZ-binding motif (TAZ) has been reported to be involved in tumor progression, angiogenesis, epithelial-mesenchymal transition (EMT), glycometabolic modulation and reactive oxygen species (ROS) buildup. Herein, the underlying molecular mechanisms of the TAZ-induced biological effects in bladder cancer were discovered.

**Methods:**

qRT-PCR, western blotting and immunohistochemistry were performed to determine the levels of TAZ in bladder cancer cells and tissues. CCK-8, colony formation, tube formation, wound healing and Transwell assays and flow cytometry were used to evaluate the biological functions of TAZ, miR-942-3p and growth arrest-specific 1 (GAS1). QRT-PCR and western blotting were used to determine the expression levels of related genes. Chromatin immunoprecipitation and a dual-luciferase reporter assay were performed to confirm the interaction between TAZ and miR-942. In vivo tumorigenesis and colorimetric glycolytic assays were also conducted.

**Results:**

We confirmed the upregulation and vital roles of TAZ in bladder cancer. TAZ-induced upregulation of miR-942-3p expression amplified upstream signaling by inhibiting the expression of large tumor suppressor 2 (LATS2, a TAZ inhibitor). MiR-942-3p attenuated the impacts on cell proliferation, angiogenesis, EMT, glycolysis and ROS levels induced by TAZ knockdown. Furthermore, miR-942-3p restrained the expression of GAS1 to modulate biological behaviors.

**Conclusion:**

Our study identified a novel positive feedback loop between TAZ and miR-942-3p that regulates biological functions in bladder cancer cells via GAS1 expression and illustrated that TAZ, miR-942-3p and GAS1 might be potential therapeutic targets for bladder cancer treatment.

**Supplementary Information:**

The online version contains supplementary material available at 10.1186/s13046-021-01846-5.

## Background

Bladder cancer is the ninth most common malignant tumor worldwide and ranks 13th in cancer-related mortality every year. There were approximately 430,000 new-onset cases in 2012 [1–4]. Bladder cancer can be classified into non-muscle-invasive and muscle-invasive bladder cancer according to the depth of tumor infiltration [5, 6]. Although timely surgical intervention and chemotherapy can restrict the progression and development of tumors, the 5-year overall survival rate of muscle-invasive bladder cancer patients is 60% due to distant metastasis [7]. Therefore, clarification of the molecular mechanisms underlying bladder cancer progression to discover novel and precise therapeutic targets and improve the prognosis of bladder cancer is meaningful.

In recent decades, numerous studies have confirmed the crucial roles of the Hippo pathway in tissue homeostasis, cell proliferation, apoptosis and multiple other biological processes. Surprisingly, impairment of the Hippo pathway leads to remarkable impacts on cancer development, angiogenesis, progression, metabolic phenotype and ROS buildup [8–15]. Moreover, emerging studies have demonstrated the regulatory roles of components of the Hippo signaling pathway in the EMT process [16–20]. EMT plays essential roles during normal mammalian development, in which epithelial cells acquire mesenchymal features. However, EMT is also associated with tumorigenesis and metastasis and is essential in cancer progression [21–23]. Therefore, EMT-related signaling pathways have been a novel focus in studies related to cancer therapy in past decades [24–27].

The core of the Hippo pathway is composed of a kinase cascade, transcriptional coactivators, and DNA-binding partners. The pathway is regulated by intrinsic cellular machinery and various cellular signals [28]. The upstream serine/threonine kinases MST1/2 (mammalian sterile twenty-like) can phosphorylate and activate LATS1/2 (large tumor suppressor) via a complex formed with the adaptor protein Sav1. Then, activated LATS1/2, together with MOB1, suppresses the transcriptional coactivator TAZ or its paralog YAP (Yes-associated protein) through phosphorylation [29]. TAZ interacts with the TEA domain DNA-binding (TEAD) family of transcription factors to recruit these transcription factors to their target promoters and regulate gene expression [30]. In mammals, the transcriptional activation of TEADs requires transcriptional coactivators, such as TAZ, YAP and the p160 family of nuclear receptor coactivators [31].

MicroRNAs regulate the levels of protein-coding genes by binding to specific mRNA sequences [32]. A growing number of studies have reported that microRNAs are involved in multiple aspects of biological cellular processes, including cancer development and progression, making them novel therapeutic targets [27, 33, 34]. Moreover, the dysregulation of miRNAs and their influences on tumorigenesis, development, and progression have been discovered in bladder cancer [35, 36].

GAS1 is a well-known cell growth suppressor [37] and is involved in tumorigenesis and progression [38–40]. Aberrant expression of GAS1 reduces tumorigenicity in human brain tumor-initiating cells [41], while downregulation of GAS1 expression is a potential biomarker of clear cell renal cell carcinoma [42]. Interestingly, GAS1 has been reported to serve as a novel biomarker and inhibit proliferation, angiogenesis, EMT and glycolysis in human cancers [43, 44].

In the current study, we investigated the abundant expression of TAZ in both bladder cancer cell lines and bladder cancer tissues. In addition, TAZ knockdown impaired proliferation, angiogenesis, EMT, glycolysis and redox homeostasis in bladder cancer cells. Mechanistically, we identified a positive feedback loop between TAZ and miR-942-3p that enhanced upstream signaling and modulated biological and metabolic phenotypes and ROS levels by regulating GAS1 expression. Collectively, our results indicate that TAZ, miR-942-3p and GAS1 are novel therapeutic targets that could be exploited for clinical intervention in bladder cancer.

## Materials and methods

### Ethical approval

All animal experiments were approved by the Ethics Committee of The First Affiliated Hospital, School of Medicine, Zhejiang University and were carried out according to the guidelines of the Guide for the Care and Use of Laboratory Animals published by the NIH.

### Clinical tissue specimens

Clinical tissue specimens and paired normal bladder tissue specimens were acquired from surgical specimens. All patients included in the study provided written informed consent. All specimens were histologically characterized by pathologists according to the World Health Organization Consensus Classification and TNM staging system for bladder neoplasms. The study was approved by the Ethics Committee of The First Affiliated Hospital of the Zhejiang University School of Medicine. Detailed information on the patients is listed in Tables S1 and S2 in Supplemental File 1.

### Cell lines and culture

SV-HUC-1 cells, HEK-293 T cells, HUVECs and the human bladder cancer cell lines 5637, J82, T24, EJ, TCCSUP, RT4 and UM-UC-3 were acquired from the American Type Culture Collection. HEK-293 T cells, J82 cells and HUVECs were cultured in Dulbecco’s modified Eagle’s medium containing 10% fetal bovine serum, and the other cells mentioned above were cultured in RPMI-1640 medium.

### RNA extraction and quantitative real-time PCR

Total RNA was extracted with TRIzol Reagent (Invitrogen, CA, USA). For mRNA detection, the PrimeScript RT Reagent Kit (Takara Bio Inc., China) was used for mRNA reverse transcription. qRT-PCR was performed by utilizing TB Green Premix Ex Taq II (Takara Bio Inc., China) with a QUANT5 PCR system (Applied Biosystems, USA). The normalized control for mRNA expression analysis was GAPDH. An All-in-One miRNA qRT-PCR detection kit (GeneCopoeia, USA) was used for miRNA detection, with human U6 as the endogenous control. The primers used were as follows: **TAZ**: Fwd, 5′-ACCCGCGAGTACAACCTTCTT-3′, and Rev., 5′-TATCGTCATCCATGGCGAACT-3′; **E-cadherin**: Fwd, 5′- CTGTGCCCAGCCTCCATGTTTT − 3′, and Rev., 5′- CTGGATAGCTGCCCATTGCAAGTTA − 3′; **N-cadherin**: Fwd, 5′- GCTTATCCTTGTGCTGATGTTT − 3′, and Rev., 5′- GTCTTCTTCTCCTCCACCTTCT − 3′; **Vimentin**: Fwd, 5′- CAGGATGTTGACAATGCGT − 3′, and Rev., 5′- CTCCTGGATTTCCTCTTCGT − 3′; **Fibronectin**: Fwd, 5′- TTATGACGACGGGAAGACCT − 3′, and Rev., 5′- GCTGGATGGAAAGATTACTC − 3′; **Snail**: Fwd, 5′- ATGCACATCCGAAGCCACA − 3′, and Rev., 5′- TGACATCTGAGTGGGTCTGG -3′; **PFKFB3**: Fwd, 5′- GTGCCTTAGCTGCCTTGAGA − 3′, and Rev., 5′- CCGACTCGATGAAAAACGCC -3′; **LDHB**: Fwd, 5′- TGGTATGGCGTGTGCTATCAG − 3′, and Rev., 5′- TTGGCGGTCACAGAATAATCTTT -3′; **HK2**: Fwd, 5′- GATTGTCCGTAACATTCTCATCGA − 3′, and Rev., 5′- TGTCTTGAGCCGCTCTGAGAT -3′; **GLUT1**: Fwd, 5′- CTTTGTGGCCTTCTTTGAAGT − 3′, and Rev., 5′- CCACACAGTTGCTCCACAT -3′; **GLUT3**: Fwd, 5′- AAAGTCCCTGAGACCCGTGGCAGG − 3′, and Rev., 5′- AAGATCCAAACCGCAGCCTTG -3′; **GLUT4**: Fwd, 5′- TGGAAGGAAAAGGGCCATGCTG − 3′, and Rev., 5′- CAATGAGGAATCGTCCAAGGATG -3′; **GAPDH**: Fwd, 5′-GATATTGTTGCCATCAATGAC-3′, and Rev., 5′-TTGATTTTGGAGGGATCTCG − 3′; **miR-942-3p**: CACATGGCCGAAACAGAGAAGT. Data analysis of relative expression levels was performed using the 2^-ΔΔCt^ method. In detail, ΔCt = Ct (target gene)-Ct (GAPDH) and ΔΔCt = ΔCt (Group A)-ΔCt (Group B).

### siRNA, plasmid and lentivirus

siRNAs and TAZ, TEAD2, miR-942 promoter and GAS1 plasmids were constructed and obtained from Transheep (Shanghai, China). A miR-942-3p mimic was synthesized by RiboBio (Guangzhou, China). siRNA was transfected with Lipofectamine™ RNAiMAX Transfection Reagent (Invitrogen, USA), while plasmids were transfected with Lipofectamine™ 3000 Transfection Reagent (Invitrogen, USA) according to the manufacturer’s instructions. Lentivirus-pre-miR-942, lentivirus-miR-942-3p-sponge and lentivirus-shTAZ were all purchased from GeneChem (Shanghai, China). Cells were transduced with lentiviruses and selected with puromycin for 1 week.

### Chromatin immunoprecipitation (ChIP)

The SimpleChIP® Plus Enzymatic Chromatin IP Kit (Catalog# 9004, Cell Signaling Technology, USA) was utilized for a ChIP assay according to the manufacturer’s instructions. Briefly, 293 T cells (4 × 10^6^) were fixed with formaldehyde and lysed, and chromatin was fragmented by digestion with Micrococcal nuclease to obtain fragments of 1–5 nucleosomes. Chromatin immunoprecipitation was performed using 2 μg antibodies and Protein G Agarose Beads with incubation overnight at 4 °C with rotation. The eluents from the immunoprecipitants were used for reversal of cross-linking. Then, we purified DNA and performed qRT-PCR with specific primers. The sequences of the primers for the miR-942 promoter were as follows: **Primer-1**: Fwd, 5′-TTTGCTCCCTTGACTCCCAGC-3′, and Rev., 5′-GGTCAAAGCACTGAGCTGTTCTT-3′; **Primer-2**: Fwd, 5′-ATTGCACTGAAGTGGGTTTTCTGT-3′, and Rev., 5′- GACACAGTCTCTAGAGTCAAGCCT-3′; **Primer-3**: Fwd, 5′-CTTCAGAGTGAGCTATTGGGCTAAAAT-3′, and Rev., 5′-CCTTCCCTACTTGAAACAACCGTATG-3′; **Primer-4**: Fwd, 5′-CCTTCAGAGTGAGCTATTGGGC-3′, and Rev., 5′-CCTTCCCTACTTGAAACAACCGT-3′; and **Primer-5**: Fwd, 5′-CCAGCCATATGAGGACAGAGGAAG-3′, and Rev., 5′-CTTTCAAGAGCCTCTAAGGGCCC-3′.

### Luciferase reporter assay

To verify the transcriptional activity of TAZ-TEAD at the miR-942 promoter, 293 T cells were plated in 96-well plates (5000 cells per well) and cotransfected with a firefly luciferase plasmid containing the miR-942 promoter, a TEAD2 plasmid and a TAZ plasmid (Transheep, China). pRL-CMV Renilla luciferase was also cotransfected to normalize luciferase activity. The ratio of TAZ plasmid: TEAD2 plasmid: miR-942 luciferase: Renilla: transfection reagent was 0.25 μg: 0.25 μg: 0.25 μg: 0.005 μg: 0.2 μL (per well), and every test was performed in triplicate. Plasmids were transfected with Lipofectamine™ 3000 Transfection Reagent (Invitrogen, USA). Cells were harvested after 48 h, and a dual-luciferase reporter assay detection kit (Promega, USA) was used for cell luciferase activity detection.

To confirm the relationships between miR-942-3p and GAS1 or LATS2, luciferase reporter vectors (pGL3-Firefly_Luciferase-Renilla_Luciferase) containing the full-length 3′-UTR of GAS1 or LATS2 were constructed, and mutant vectors were also generated (GeneChem, China). Two hundred ninety-three T cells were seeded and cotransfected with a luciferase vector and miR-942-3p mimic or negative control. The cells were harvested after 48 h, and a dual-luciferase reporter assay detection kit (Promega, USA) was used to measure firefly and Renilla luciferase activities. The final result = firefly luciferase intensity/Renilla luciferase intensity. All assays were performed independently at least three times.

### Tube formation assay

HUVECs were seeded in a 96-well plate (2 × 10^4^ per well) precoated with Matrigel (BD Biosciences, USA). Conditioned medium (CM) acquired from different cells was added into the wells, and the plate was incubated for 6 h. The formation of tubes was observed by phase-contrast microscopy (Nikon, Japan) and quantified by ImageJ in three randomly selected fields.

### Migration and invasion assays

Migration and invasion chambers (Costar, NY, USA) were used in migration and invasion assays. Briefly, 3 × 10^4^ cells in serum-free medium were seeded in the upper chambers. Specifically for the invasion assay, upper chambers precoated with Matrigel (BD Biosciences, USA) were used. Medium containing 10% fetal bovine serum was added to the bottom chambers. Migrated and invaded cells were fixed with 4% formalin for 15 min and stained with crystal violet for another 15 min. The stained cells were observed and counted by microscopy (Nikon, Japan) in three randomly selected fields.

### Wound healing assay

Seventy microliters of bladder cancer cell suspension was plated in a well containing Culture-Insert (Ibidi, Germany) according to the manufacturer’s instructions. After 24 h, Culture-Inserts were removed, and the cells were incubated in serum-free medium. Photos were captured at 0 and 24 h after insert removal, and the migration rate of the cells was measured and analyzed with ImageJ software.

### Apoptosis analysis

Cells under different conditions were seeded in a 6-well plate. After 24 h of incubation, the cells were collected and stained with an Annexin V-FITC/propidium iodide (PI) kit (BD Biosciences, USA). After an incubation for 15 min at room temperature, the apoptotic rate of the cells was detected by flow cytometry (Becton Dickinson, USA).

### Detection of intracellular ROS

Intracellular ROS levels were determined by using DCFH–DA (Sigma, MO, USA). Specifically, cells that received various treatments were incubated with DCFH–DA (5 μM) in serum-free medium for 30 min at 37 °C. After washing three times with PBS, the level of ROS was detected and analyzed by flow cytometry (Becton Dickinson, USA).

### Western blot analysis and antibodies

Total protein was by using RIPA buffer (C1053, APPLYGEN, Beijing, China), and the protein concentration was determined by using a BCA protein assay kit (Beyotime, China). Proteins were then separated by SDS-PAGE and transferred to PVDF membranes (Millipore, Billerica, MA). After blocking for 2 h in 5% milk, membranes were washed with TBST 3 times and incubated with primary antibodies at 4 °C overnight. The membranes were then incubated with a secondary antibody (anti-mouse or anti-rabbit IgG, Cell Signaling Technology, USA) for 1 h at room temperature. After 3 washes with TBST, a Bio-Rad detection system was used to detect the bands. The antibodies used in this study were as follows: anti-GAPDH (5174), anti-TAZ (70148), anti-E-cadherin (3195), anti-Snail (3879), anti-N-cadherin (13116), anti-Vimentin (5741) and anti-Fibronectin (26836) were all obtained from Cell Signaling Technology. Anti-LATS2 (ab110780), anti-GAS1 (ab236618), anti-PFKFB3 (ab181861), anti-HK2 (ab209847) and anti-GLUT1 (ab115730) were all obtained from Abcam.

### Glycolysis process evaluation

The uptake of glucose into different cells was evaluated with the Glucose Uptake Colorimetric Assay Kit (BioVision, CA, USA). In detail, 1 × 10^4^ cells per well were seeded in a 96-well plate. To evaluate glucose uptake, the cells were starved in 100 μl serum-free medium overnight and then preincubated with 100 μl Krebs-Ringer-Phosphate-Hepes (KRPH) buffer containing 2% BSA for 40 min. Then, 10 μl 10 mM 2-DG was added and incubated for 20 min. The cells were lysed to degrade endogenous NAD(P) and denature enzymes and then heated at 85 °C for 40 min. The cell lysates were cooled on ice for 5 min. Finally, the absorbance was measured by using a microplate reader. The production of lactate was detected by using a lactate colorimetric assay kit (BioVision, CA, USA). Briefly, cells were homogenized in assay buffer. After centrifugation to remove insoluble materials, 50 μl was added to each well of a 96-well plate. Fifty microliters of reaction mix was added to each well, and the plate was incubated for 30 min at room temperature. The absorbance was measured by using a microplate reader, and the lactate concentration was calculated.

### Other in vitro experiments

CCK-8 and colony formation assays and HE staining were performed according to previously described methods [27].

### In vivo studies

To establish a xenograft tumorigenesis model, 5 × 10^6^ T24 cells that received different treatments were injected subcutaneously into nude mice. The mice were sacrificed, and the tumors were excised, weighed, and fixed with formalin for immunohistochemical examination 25 days later. Tumor volume was measured daily and calculated according to the following formula: Total tumor volume (mm^3^) = L × W^2^/2 (“L” = length and “W” = width). All animal-related procedures were approved by the Animal Care and Use Committee of The First Affiliated Hospital of the School of Medicine of Zhejiang University.

### Statistical analysis

All data are expressed as the mean ± standard deviation (SD) and were analyzed with SPSS. A paired t-test was used to analyze differential expression in both cells and tissues. A two-tailed *p*-value < 0.05 was regarded as statistically significant in this study.

## Results

### TAZ expression is markedly elevated in bladder cancer cell lines and tissue

**Fig. 1:**
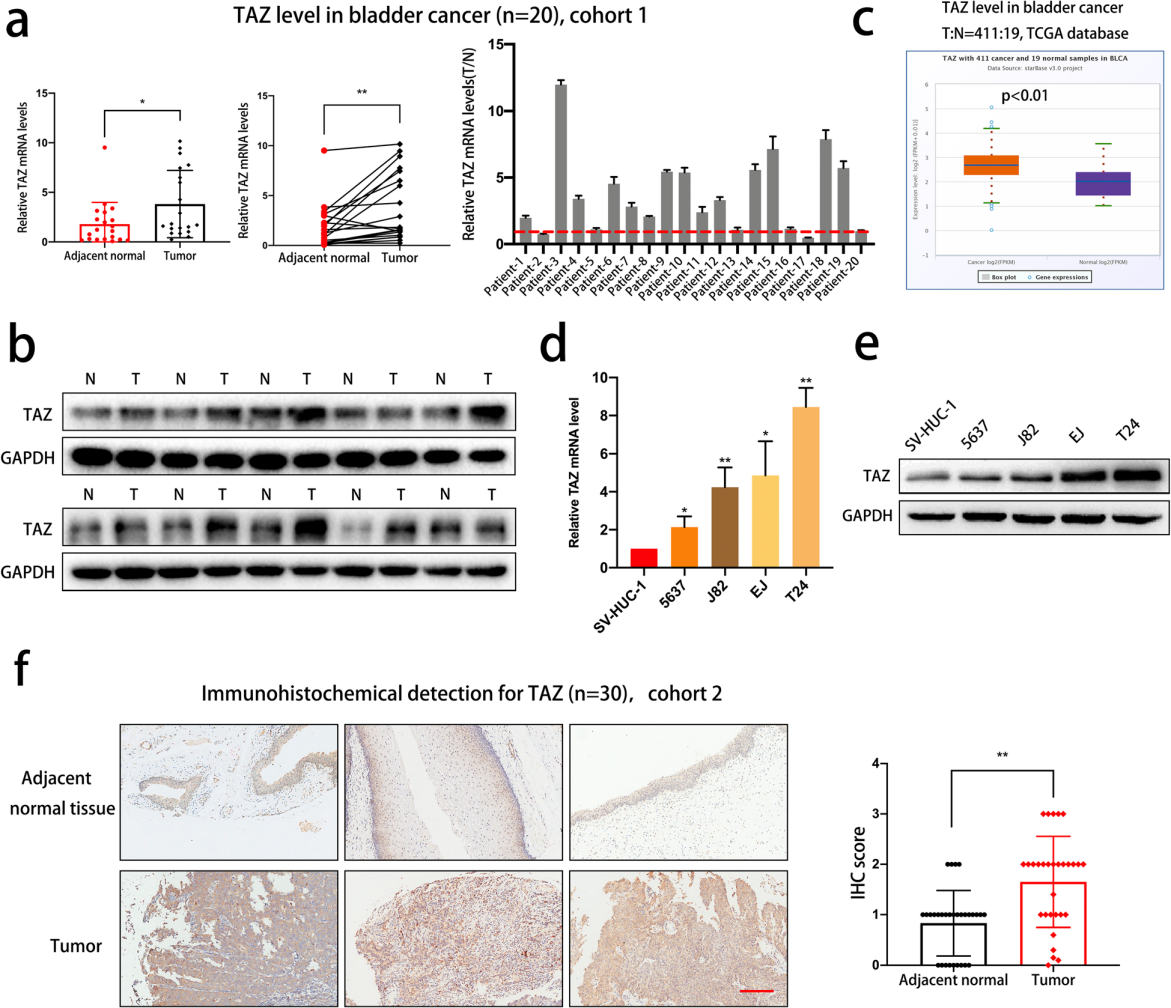
TAZ is overexpressed in bladder cancer cell lines and tissues. **a-b** Detection of TAZ expression in human bladder cancer tissues and adjacent normal tissues at the mRNA and protein levels (cohort 1, *n* = 20). **c**. TAZ expression levels in bladder cancer tissue and normal tissue in the TCGA database. **d-e** mRNA and protein expression levels of TAZ in SV-HUC-1 cells and different bladder cancer cell lines. **f**. The expression levels of TAZ in bladder cancer tissue specimens in cohort 2 were determined by IHC (*n* = 30). TAZ expression was significantly upregulated in bladder cancer tissue. Scale bar: 200 μm. Data are presented as the mean ± SD of three independent experiments. *P < 0.05 and **P < 0.01 vs. the control group

First, we investigated TAZ expression differences between human bladder cancer tissues and matched adjacent normal tissues (cohort 1, *n* = 20). As shown in Fig. 1a and b, TAZ was overexpressed in the bladder cancer tissues at both the mRNA and protein levels. Additionally, data from the TCGA database produced a similar trend (Fig. 1c). We then detected TAZ levels in SV-HUC-1 cells and bladder cancer cell lines. The results revealed that TAZ expression was markedly upregulated in the bladder cancer cell lines compared with the normal human uroepithelial cells (Fig. 1d and e). Furthermore, we performed an immunohistochemical assay and found that TAZ expression was higher in bladder cancer tissue than in adjacent normal tissue in cohort 2 (*n* = 30, Fig. 1f).

### TAZ knockdown suppresses proliferation, colony formation, angiogenesis, migration, invasion and mesenchymal transformation in bladder cancer cells

**Fig. 2:**
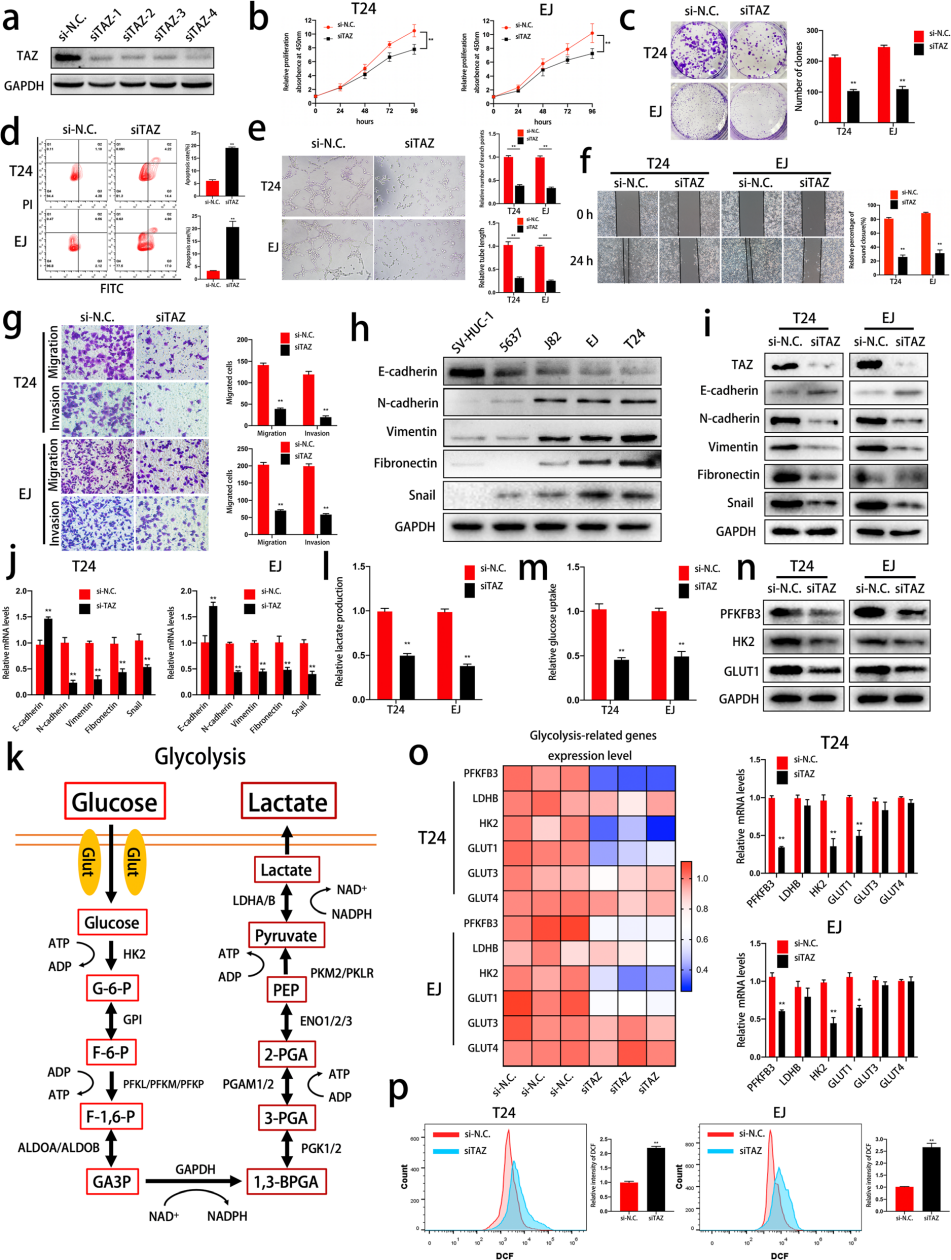
TAZ is vital in cell growth, angiogenesis, migration, invasion, EMT, glycolysis and ROS homeostasis in bladder cancer cells. **a.** The knockdown efficiency of siRNAs specific for TAZ was determined by western blot analysis of T24 cells. **b.** A CCK-8 assay was performed to determine the effect of TAZ on cell viability. **c.** Depletion of TAZ inhibited the colony-forming abilities of T24 and EJ cells. **d.** The apoptotic rates of control and TAZ knockdown cells were detected by flow cytometry. **e.** A tube formation assay was performed to evaluate the effect of TAZ on tumor angiogenesis. **f.** A wound healing assay was used to evaluate the cell migration of control or TAZ-knockdown T24 and EJ cells. **g.** The cell migration and invasion of TAZ-deficient cells were evaluated by Transwell migration and invasion assays, respectively. **h.** EMT markers were detected in SV-HUC-1 cells and bladder cancer cell lines by western blotting. **i-j** The key role of TAZ in the EMT process was confirmed by western blotting and qRT-PCR. **k.** A schematic illustration of glycolysis is shown. **l-m** The uptake of glucose and production of lactate in TAZ-deficient and normal bladder cancer cells were determined. **n.** The alterations in PFKFB3, HK2 and GLUT1 at the protein level were confirmed by western blotting. **o.** qRT-PCR was performed to assess the expression levels of glycolysis-related genes (PFKFB3, LDHB, HK2, GLUT1, GLUT3 and GLUT4). **p.** Intracellular ROS levels were determined with DCFH–DA and analyzed by flow cytometry. Data are presented as the mean ± SD of three independent experiments. *P < 0.05 and **P < 0.01 vs. the control group

To evaluate the potential roles of TAZ in bladder cancer cells, we used a short interfering RNA (siRNA) to deplete TAZ. SiRNA efficiency was evaluated by western blot analysis of T24 cells (Fig. 2a). CCK-8 and colony formation assays demonstrated that TAZ knockdown suppressed cell proliferation and colony formation (Fig. 2b and c). We also detected the apoptotic rate by flow cytometry and found that significant apoptosis was triggered by depletion of TAZ (Fig. 2d). Moreover, a tube formation assay indicated that TAZ played a key role in tumor angiogenesis (Fig. 2e). A wound healing assay indicated that TAZ was involved in bladder cancer cell migration (Fig. 2f). In addition, depletion of TAZ significantly decreased the numbers of migrated T24 and EJ cells in Transwell migration and Matrigel invasion assays (Fig. 2g). Previous studies have reported that cell migration and EMT are closely associated with tumor migration, invasion, progression and metastasis [21–23, 25]. Interestingly, we found that TAZ expression was positively correlated with that of mesenchymal markers (Vimentin, N-cadherin, Fibronectin and Snail) and negatively correlated with that of an epithelial marker (E-cadherin) (Fig. 2h). The above results indicated a key role for TAZ in the EMT process. As we predicted, TAZ inhibition led to downregulation of N-cadherin, Vimentin, Fibronectin and Snail and upregulation of E-cadherin at the protein and mRNA levels (Fig. 2i and j). Our current results demonstrate that TAZ serves as a vital regulator of various biological functions in bladder cancer cells.

### Inhibition of TAZ suppresses glycolysis by regulating PFKFB3, HK2 and GLUT1 expression in bladder cancer cells

Numerous studies have reported that the Hippo pathway is involved in glucose metabolism [12, 13]. Furthermore, a specific glycolysis-dependent metabolic phenotype is well accepted as a characteristic of tumor cells. Therefore, we evaluated the role of TAZ in the glycometabolism of bladder cancer cells. Figure 2k illustrates the process of aerobic glycolysis. As shown in Fig. 2l and m, inhibition of TAZ significantly decreased the uptake of glucose and production of lactate, which indicated that glycolysis was suppressed. In addition, we also detected the expression levels of several glycolysis-related genes to clarify the underlying regulatory mechanism involving TAZ in the regulation of glycolysis. The results demonstrated that TAZ knockdown decreased PFKFB3, HK2 and GLUT1 at the mRNA and protein levels (Fig. 2n and o). These results illustrated that TAZ might function as a key factor in aerobic glycolysis in bladder cancer.

### TAZ inhibition leads to ROS dysregulation in bladder cancer

A previous study indicated that TAZ deficiency induced a transition in the metabolic phenotype and ROS accumulation in NF2-mutant tumor cells, rendering tumor cells more vulnerable under nutrient stress [14]. In addition, our previous study reported that ROS were closely related to cell death and survival in bladder cancer [27]. In light of this evidence, we used DCFH-DA, a sensitive fluorescence probe for ROS, to evaluate ROS stress in TAZ-knockdown bladder cancer cells. As shown in Fig. 2p, depletion of TAZ remarkably increased the level of ROS, which suggests that TAZ plays a vital role in intracellular ROS homeostasis.

### MiR-942-3p is regulated by TAZ-TEAD

**Fig. 3:**
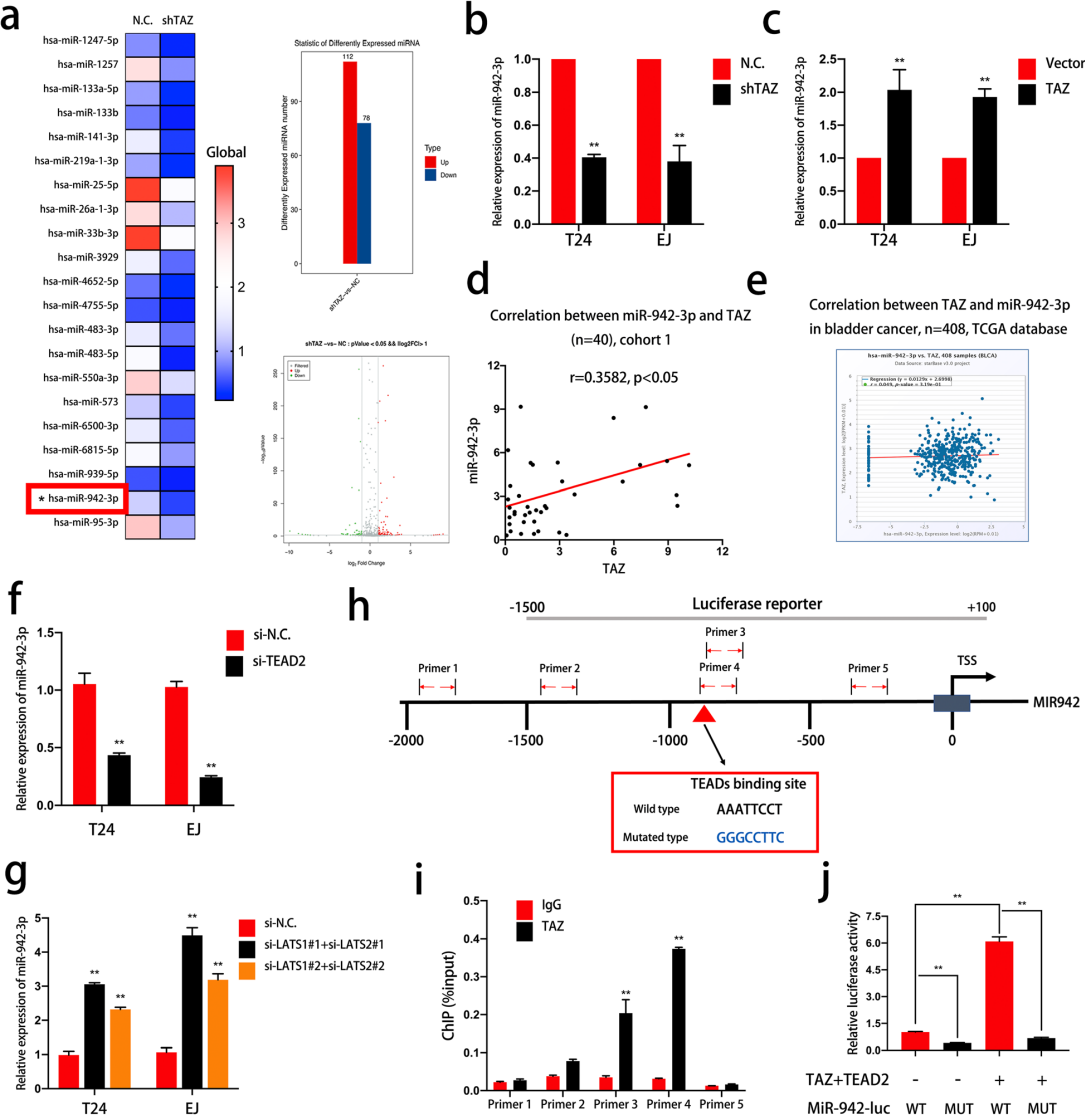
TAZ-TEAD regulates the level of miR-942-3p by binding to its promoter. **a.** TAZ-related miRNAs in T24 cells were identified by RNA sequencing. A heatmap of these miRNAs was generated (N.C. vs shTAZ cells, each sample was mixed with three replicates). **b-c** qRT-PCR detection of miR-942-3p in TAZ-knockdown or TAZ-overexpressing cells. **d-e** Correlation between TAZ and miR-942-3p in bladder cancer in cohort 1 and the TCGA database. **f-g** The Hippo signaling pathway induces miR-942-3p expression. MiR-942-3p levels in LATS1/2-deficient or TEAD2-deficient cells were determined by qRT-PCR. **h.** Schematic illustration of the miR-942 promoter. Predicted TEAD binding site in the miR-942 promoter (indicated by the red arrow). The section of the promoter cloned for a luciferase reporter assay and the primers used for qRT-PCR validation in a ChIP assay are indicated. **j.** Chromatin immunoprecipitation followed by qRT-PCR using specific primers for different regions of the miR-942 promoter in 293 T cells demonstrated that TAZ could bind to the miR-942 promoter. **k.** The predicted TEAD binding site was mutated, and a luciferase reporter assay was performed to validate the binding site of TAZ-TEADs in the miR-942 promoter. Data are presented as the mean ± SD of three independent experiments. *P < 0.05 and **P < 0.01 vs. the control group

To clarify the regulatory mechanism involving TAZ in bladder cancer, we verified miRNAs regulated by TAZ in T24 bladder cancer cells by RNA sequencing (Fig. 3a and Supplemental File 2). Among the miRNAs downregulated by TAZ knockdown, miR-942-3p was reported to be a key factor in cell proliferation and invasion [45, 46], and we selected miR-942-3p as our research subject. qRT-PCR was used to confirm the remarkable downregulation of miR-942-3p expression in TAZ-knockdown bladder cancer cells (Fig. 3b). Furthermore, TAZ overexpression enhanced miR-942-3p expression (Fig. 3c). In addition, the expression levels of TAZ and miR-942-3p were positively correlated in both cohort 1 and the TCGA database (Fig. 3d and e). We also found alterations in miR-942-3p in LATS1- and LATS2- or TEAD2-knockdown cells, which indicated that the Hippo signaling pathway plays a key role in the TAZ/miR-942-3p regulatory mechanism (Fig. 3f and g). TAZ interacts with TEAD to recruit TEAD to its target promoters and regulate gene expression [30, 31, 47]. Therefore, we performed ChIP to investigate the interaction between TAZ and the miR-942 promoter. qRT-PCR evaluation of the miR-942 promoter was performed with different primers (indicated in Fig. 3h). As shown in Fig. 3i, TAZ could enrich DNA segments. According to the above results, we then predicted the binding site of TEADs in the promoter region of miR-942 in JASPAR (Fig. 3h). A luciferase reporter assay further confirmed that the ability of TAZ-TEAD2 to activate the promoter of miR-942 depended on the presence of the TEAD-binding sequence (Fig. 3j). In summary, these results verify that the miR-942 promoter is a direct target of TAZ-TEAD.

### MiR-942-3p acts as a tumor promoter and suppresses ROS buildup in bladder cancer

**Fig. 4:**
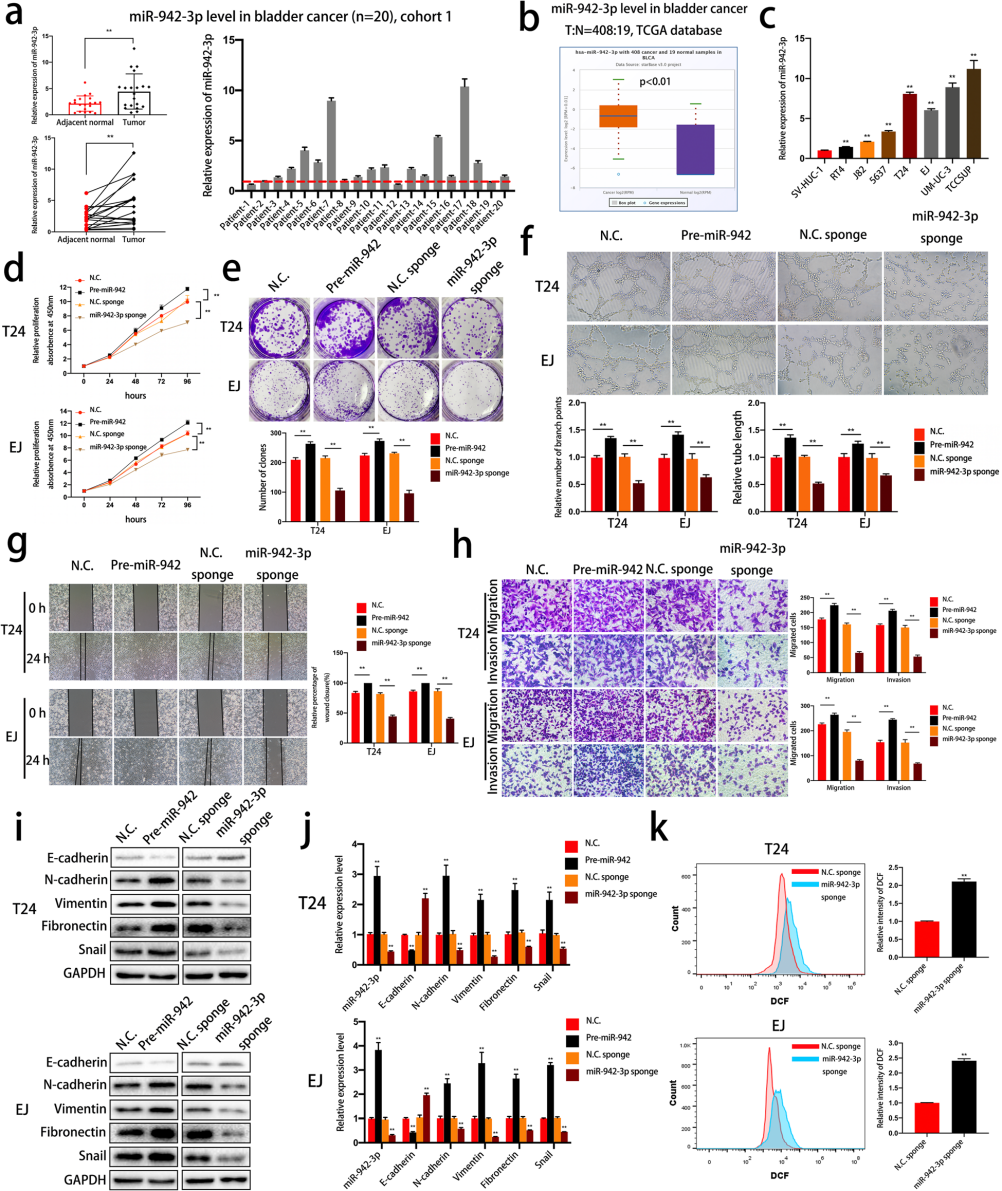
MiR-942-3p is involved in the proliferation, angiogenesis, migration, invasion, EMT process and redox balance of bladder cancer cells. **a.** MiR-433-3p was detected in bladder cancer tissue and adjacent normal tissue (cohort 1, *n* = 20). **b.** TCGA database analysis indicated the upregulation of miR-942-3p expression in bladder cancer. **c.** qRT-PCR was performed to determine miR-942-3p levels in SV-HUC-1 cells and bladder cancer cell lines. **d.** Cell viability was measured to evaluate the biological effects of miR-942-3p. **e.** The effect of miR-942-3p on colony formation was evaluated with a colony formation assay. **f.** HUVECs were cultured with conditioned medium to assess angiogenesis mediated by miR-942-3p. **g.** A wound healing assay was performed to evaluate cell migration. **h.** MiR-942-3p influenced cell migration and invasion, as determined by Transwell migration and Matrigel invasion assays, respectively. **i-j** The protein and mRNA expression levels of EMT markers in T24 and EJ cells transfected with pre-miR-942 or a miR-942-3p sponge were detected by western blotting and qRT-PCR, respectively. **k.** ROS content was evaluated by flow cytometry in different cells. Data are presented as the mean ± SD of three independent experiments. *P < 0.05 and **P < 0.01 vs. the control group

We first assessed the expression level of miR-942-3p in bladder cancer cells and tissues. The results confirmed the upregulation of miR-942-3p expression in bladder cancer tissues (cohort 1) and cell lines (Fig. 4a-c), which indicated that miR-942-3p is associated with tumorigenesis and progression in bladder cancer. To verify this hypothesis, we transfected T24 and EJ cells with pre-miR-942 or a miR-942-3p sponge. CCK-8 and colony formation assays revealed that miR-942-3p overexpression enhanced cell viability and proliferation, while miR-942-3p inhibition produced the opposite results (Fig. 4d and e). A tube formation assay indicated that miR-942-3p promoted angiogenesis in bladder cancer (Fig. 4f). In addition, wound healing, Transwell migration and Transwell invasion assays were performed to evaluate migratory and invasive abilities. The results showed that miR-942-3p acted as a promoter of bladder cancer cell migration and invasion (Fig. 4g and h). We also detected EMT markers in miR-942-3p-overexpressing or miR-942-3p-knockdown cells by western blotting and qRT-PCR to investigate whether the EMT process is regulated by miR-942-3p. As shown in Fig. 4i and j, miR-942-3p enhanced the expression of E-cadherin and suppressed the expression of N-cadherin, Vimentin, Fibronectin and Snail at the protein and mRNA levels. Conversely, miR-942-3p inhibition produced the opposite effects in bladder cancer cells. Moreover, sponging miR-942-3p led to elevation of the intracellular ROS level (Fig. 4k). In summary, our data demonstrate that miR-942-3p is a TAZ-induced and abundantly expressed miRNA in bladder cancer that plays significant roles in cell proliferation, angiogenesis, migration, invasion, EMT and redox homeostasis.

### GAS1 and LATS2 act as direct targets of miR-942-3p in bladder cancer

**Fig. 5:**
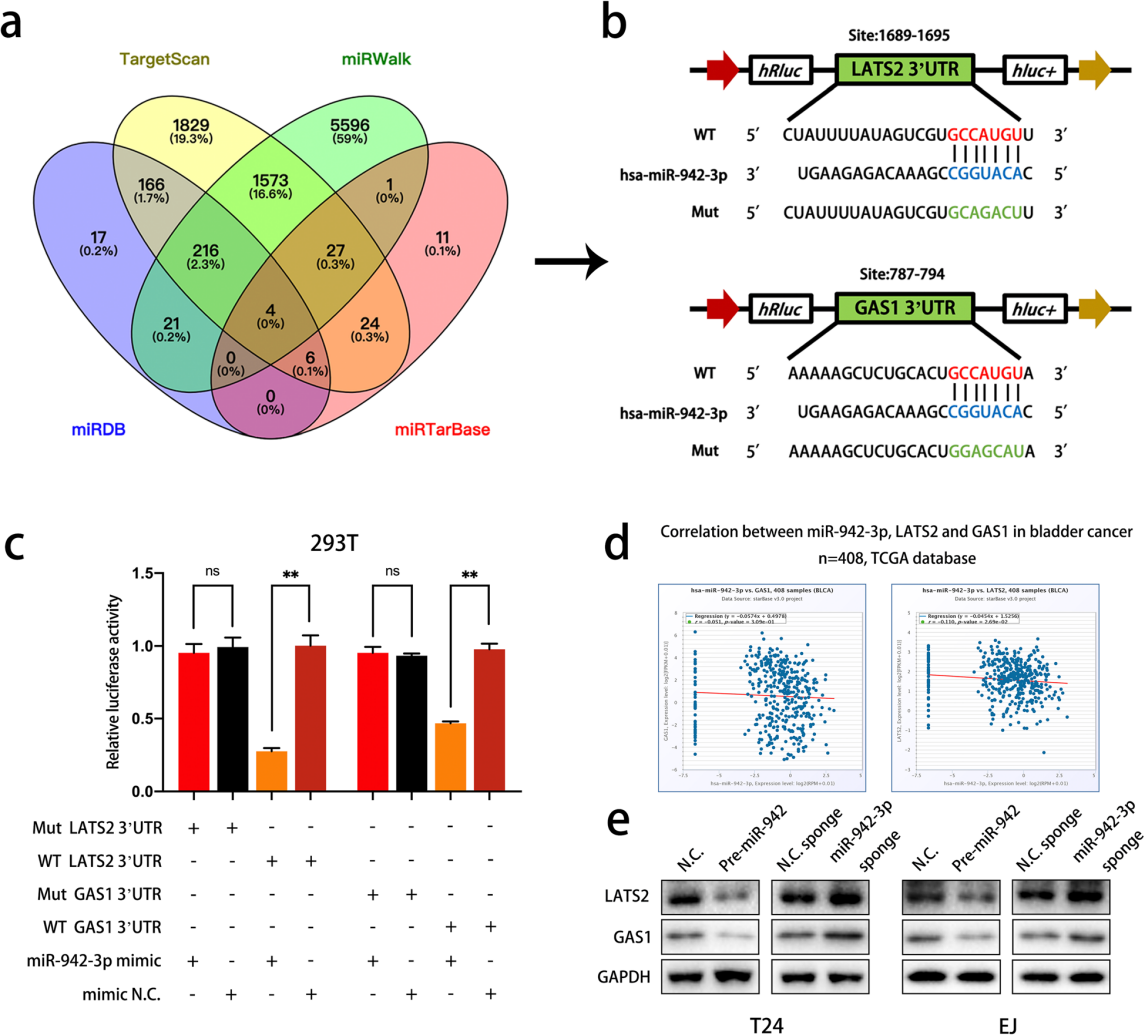
Validation of GAS1 and LATS2 as direct targets of miR-942-3p. **a.** MiR-942-3p target genes predicted by searching the TargetScan, miRDB, miRWalk and miRTarBase databases. **b.** The potential miR-942-3p binding sites in the 3′-UTRs of GAS1 and LATS2 mRNA transcripts are shown in the schematic illustration. Lowercase letters indicate the mutated binding sites in the same 3′-UTR. **c.** Luciferase reporter assay analysis of 293 T cells transfected with the indicated plasmids and miR-942-3p mimics (100 nM). **d.** TCGA database analysis indicated negative correlations among miR-942-3p, GAS1 and LATS2. **e.** The miR-942-3p level was negatively correlated with the protein levels of GAS1 and LATS2. Data are presented as the mean ± SD of three independent experiments. *P < 0.05 and **P < 0.01 vs. the control group

We then identified the molecular mechanism involving miR-942-3p in bladder cancer by predicting the target genes of miR-942-3p in several databases (TargetScan, miRWalk, miRDB and miRTarBase). First, the four lists of genes were overlapped, and four genes were identified to be targeted by miR-942-3p in all the databases (Fig. 5a). GAS1 is a well-known cell growth suppressor and is involved in tumorigenesis and progression [41–43, 48]. Interestingly, apart from the above 4 genes, we found that LATS2, a significant component of the Hippo signaling pathway, was indicated to be targeted by miR-942-3p in TargetScan. Therefore, GAS1 and LATS2 were selected for further verification. The potential binding sites of miR-942-3p within the 3′-untranslated regions (3′-UTRs) of GAS1 and LATS2 are shown in Fig. 5b. To validate whether miR-942-3p binds to these 3′-UTRs, we constructed luciferase reporter plasmids containing the full-length 3′-UTR of GAS1 or LATS2 or a corresponding mutant 3′-UTR and cotransfected them with miR-942-3p mimics. The miR-942-3p mimics induced a remarkable reduction in the luciferase activity of the wild-type GAS1 and LATS2 3′-UTRs. In addition, mutating the above 3′-UTRs eliminated the reductions in the luciferase activity of the wild-type 3′-UTRs induced by the miR-942-3p mimics (Fig. 5c). Furthermore, GAS1 and LATS2 were negatively correlated with miR-942-3p in the TCGA database and bladder cancer cell lines at the mRNA and protein levels, respectively (Fig. 5d and e). These findings demonstrate that miR-942-3p specifically targets GAS1 and LATS2.

### A TAZ/miR-942-3p positive feedback loop regulates the oncogenic effects and metabolic and ROS homeostasis modulation mediated by TAZ

**Fig. 6:**
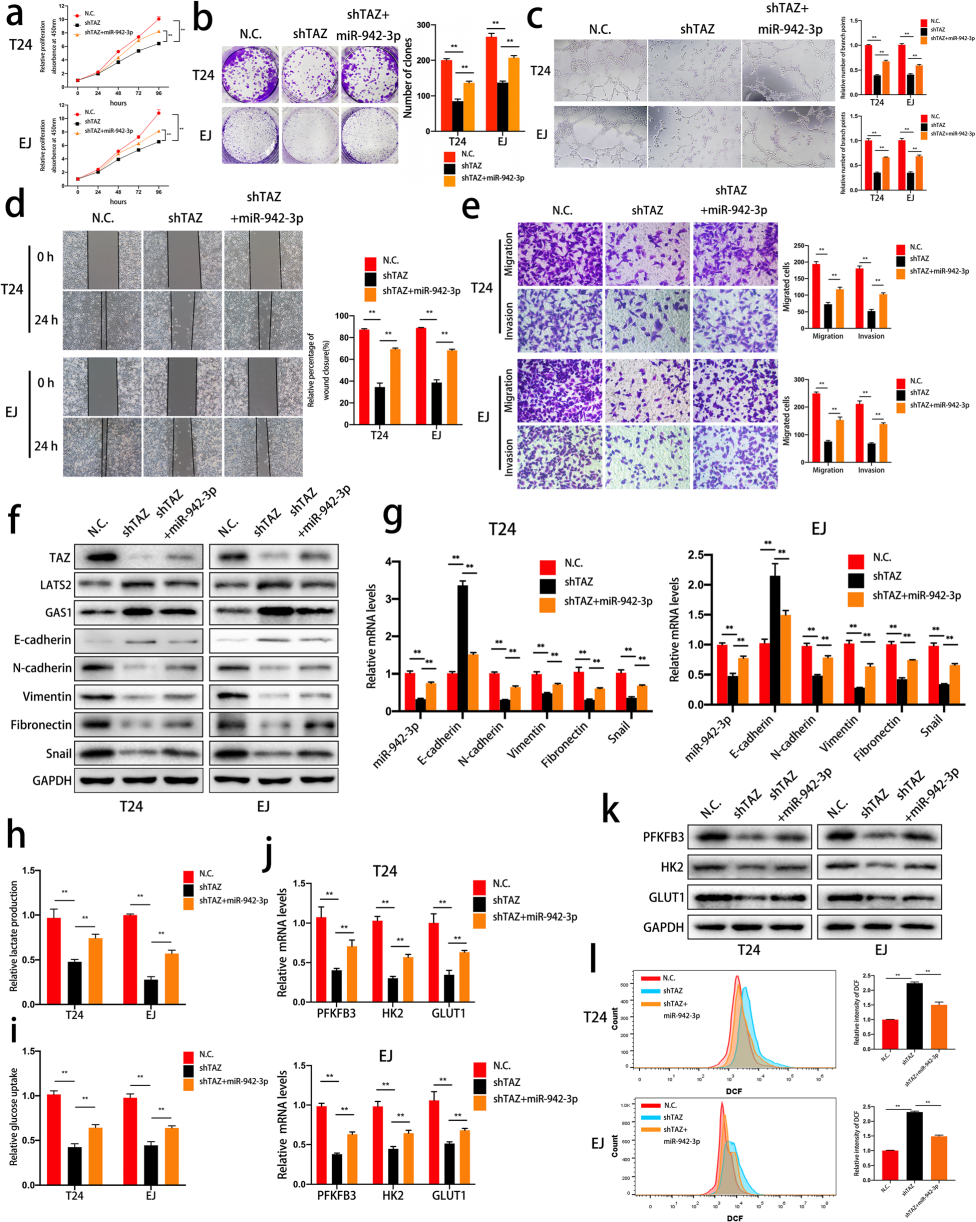
A TAZ/miR-942-3p positive feedback loop modulates the oncogenic effects, glycolysis and redox state mediated by TAZ. **a.** Cells were transfected with a negative control vector, TAZ-specific shRNA or pre-miR-942. Cell viability was determined by a CCK-8 assay. **b.** MiR-942-3p promoted colony formation, as determined by a colony formation assay. **c.** A tube formation assay was used to verify the effect of the positive feedback loop on angiogenesis. **d.** To verify the effects on cell migration, a wound healing assay was performed. **e.** The migration and invasion of bladder cancer cells transfected with different vectors were evaluated with Transwell migration and invasion assays, respectively. **f-g** The expression levels of TAZ and EMT markers in T24 and EJ cells were evaluated by western blotting and qRT-PCR. **h-i** The uptake of glucose and production of lactate in cells under different conditions were determined to evaluate the glycolytic process. **j-k** The expression levels of glycolysis-related genes (PFKFB3, HK2 and GLUT1) were detected by qRT-PCR and western blotting in TAZ-deficient cells with or without supplementation with miR-942-3p. **l.** ROS levels were detected in cells transfected with a negative control vector, TAZ-specific shRNA or pre-miR-942. Data are presented as the mean ± SD of three independent experiments. *P < 0.05 and **P < 0.01 vs. the control group

Since LATS2 inhibits the activity of TAZ in the Hippo signaling pathway, we speculated that TAZ and miR-942-3p might form a positive feedback loop in bladder cancer. To verify this regulatory mechanism, we first investigated the biological functions of TAZ-knockdown cells with or without miR-942-3p overexpression. Notably, upregulation of miR-942-3p expression partly attenuated the suppression of cell viability and proliferation induced by TAZ depletion (Fig. 6a and b). In addition, we performed a tube formation assay and found that miR-942-3p could reverse the suppression of angiogenesis induced by TAZ knockdown in HUVECs (Fig. 6c). Transwell migration, Matrigel invasion and wound healing assays indicated that miR-942-3p rescued migration and invasion in TAZ-depleted cells (Fig. 6d and e). Additionally, alterations in E-cadherin, Vimentin, N-cadherin, Fibronectin and Snail expression at the protein and mRNA levels demonstrated that the EMT process was reversed by miR-942-3p expression in TAZ knockdown cells (Fig. 6f and g). Moreover, the miR-942-3p-mediated expression levels of LATS2, GAS1 and TAZ are also shown in Fig. 6f. We then verified whether miR-942-3p can reverse the metabolic phenotype induced by TAZ knockdown. The results of glucose uptake and lactate production assays showed that glycolysis was enhanced by miR-942-3p in TAZ-deficient cells (Fig. 6h and i). PFKFB3, HK2 and GLUT1 expression was also upregulated by miR-942-3p at the mRNA and protein levels (Fig. 6j and k). Finally, ROS levels were detected by flow cytometry, and the results were consistent with those of previous assays (Fig. 6l). In summary, our results showed that a positive feedback loop between TAZ and miR-942-3p was involved in the biological functions, EMT process, glycometabolism and ROS balance of bladder cancer cells.

### GAS1 impaired the aggressive characteristics, glycolysis and ROS balance of bladder cancer cells

**Fig. 7:**
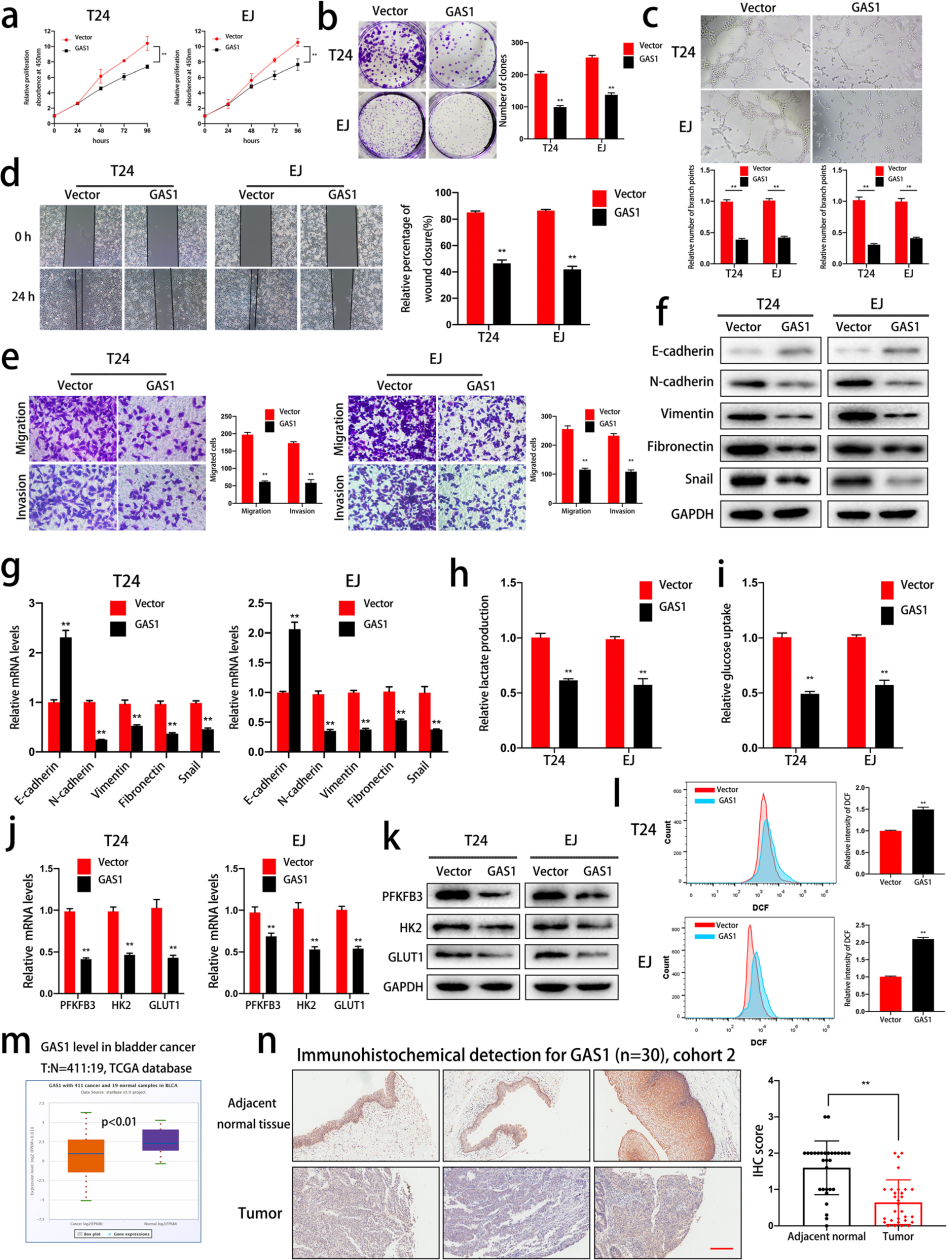
GAS1 overexpression impaired cell proliferation, angiogenesis, migration, invasion, EMT, glycolysis and ROS homeostasis in bladder cancer cells. **a.** The effect of GAS1 on cell viability was verified by a CCK-8 assay. **b.** A colony formation assay showed that GAS1 impaired the colony-forming ability of T24 and EJ cells. **c.** Conditioned medium was collected from control or GAS1-overexpressing cells and used in a tube formation assay to evaluate angiogenesis. **d.** A wound healing assay indicated the effect of GAS1 overexpression on migration. **e.** GAS1 overexpression suppressed the migration and invasion of T24 and EJ cells, as determined by Transwell migration and Matrigel invasion assays. **f-g** Western blot and qRT-PCR analyses showing the expression levels of EMT markers in GAS1-overexpressing bladder cancer cells at the protein and mRNA levels, respectively. **h-i** The effects of GAS1 on glycolysis were determined by glucose uptake and lactate production assays. **j-k** PFKFB3, HK2 and GLUT1 levels in GAS1-overexpressing cells were evaluated by western blotting and qRT-PCR. **l.** GAS1 remarkably upregulated intracellular ROS levels in T24 and EJ cells. **m.** TCGA database analysis showed a lower expression level of GAS1 expression in bladder cancer tissue than in normal tissue. **n.** Immunohistochemical detection of GAS1 in cohort 2 (*n* = 30) further confirmed the dysregulation of GAS1 in bladder cancer. Scale bar: 200 μm. Data are presented as the mean ± SD of three independent experiments. *P < 0.05 and **P < 0.01 vs. the control group

We assessed the effects of GAS1 on bladder cancer cells by constructing stable GAS1-overexpressing cells via lentiviral transduction. CCK-8 and colony formation assays showed that GAS1 inhibited the viability and proliferation of bladder cancer cells (Fig. 7a and b). A tube formation assay indicated that GAS1 inhibited tumor angiogenesis in T24 and EJ cells (Fig. 7c). Moreover, GAS1 overexpression significantly reduced the relative percentage of wound closure (Fig. 7d). Transwell migration and invasion assays further reflected the attenuation of migration and invasion induced by GAS1 overexpression in T24 and EJ cells (Fig. 7e). To assess whether GAS1 suppresses migration and invasion by regulating the EMT process, we assessed EMT marker expression at the protein and mRNA levels. The results indicated that overexpression of GAS1 enhanced E-cadherin expression and decreased the levels of Vimentin, N-cadherin, Fibronectin and Snail (Fig. 7f and g). Furthermore, detection of glucose uptake and lactate production showed that GAS1 inhibited cellular glycolysis (Fig. 7h and i). qRT-PCR and western blotting indicated that PFKFB3, HK2 and GLUT1 were downregulated at the mRNA and protein levels (Fig. 7j and k), which was consistent with previous results induced by TAZ inhibition. Additionally, upregulated ROS levels were also observed in GAS1-overexpressing cells (Fig. 7l). In addition, both TCGA database analysis and immunohistochemical detection of GAS1 in cohort 2 demonstrated GAS1 dysregulation in bladder cancer (Fig. 7m and n).

### TAZ and miR-942-3p promote the growth of xenograft tumors in vivo

**Fig. 8:**
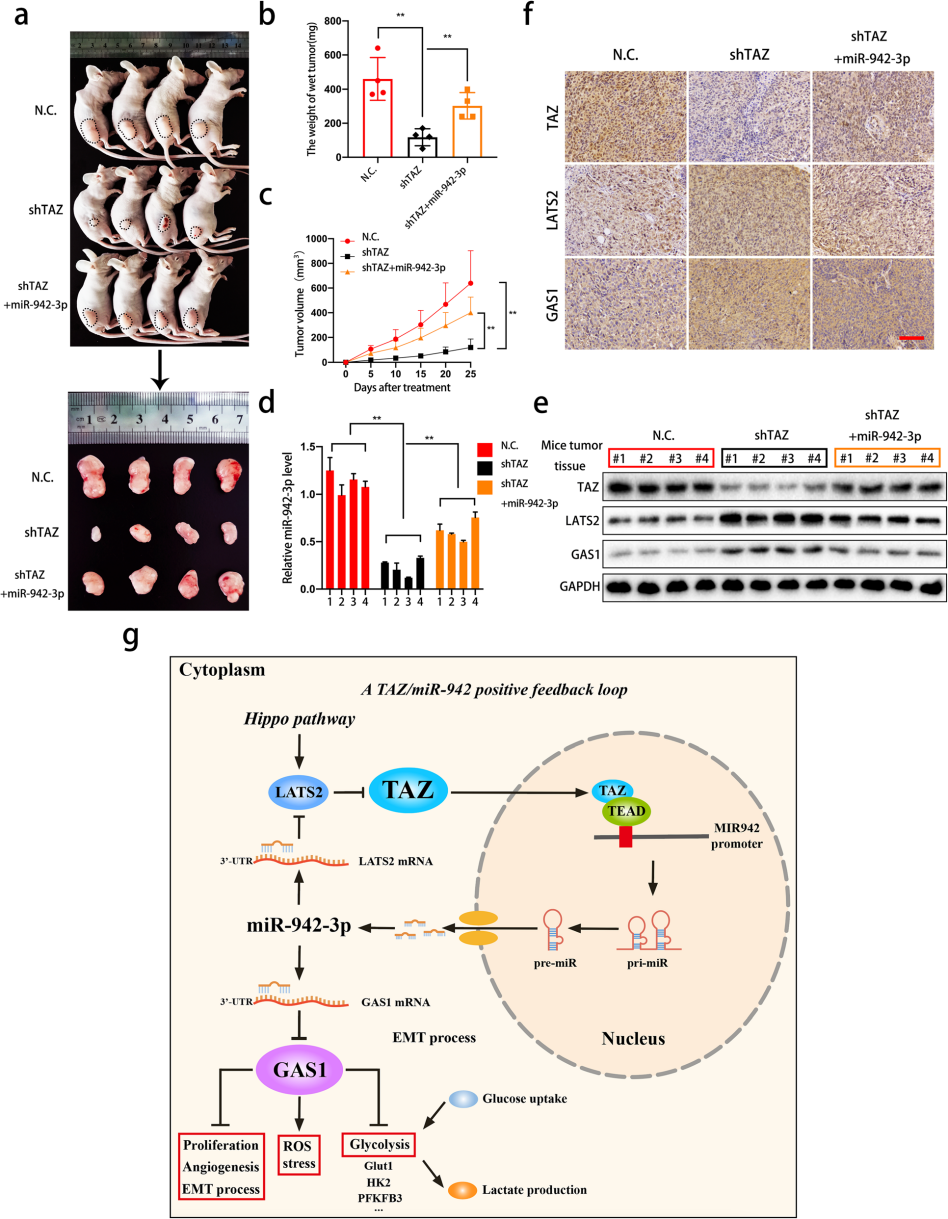
TAZ and miR-942-3p enhance bladder cancer tumor growth in vivo. **a.** Equal numbers of different T24 cell populations (negative control, shTAZ or cotransfected with shTAZ and miR-942-3p) were used to establish subcutaneous xenograft tumors. The tumors were harvested and photographed (*n* = 4 each group). **b.** Tumor weight was determined when the mice were sacrificed. **c.** Tumor volume was measured and calculated after cells were injected into mice. **d.** MiR-942-3p levels were determined by qRT-PCR in tumors obtained from mice. **e.** TAZ, LATS2 and GAS1 were detected in mouse tumor tissues by western blotting. **f.** Immunohistochemical (IHC) detection of TAZ, LATS2 and GAS1 in tumors. Scale bars: 200 μm. **g.** Schematic diagram illustrating the novel positive feedback loop between TAZ and miR-942-3p that regulates GAS1 expression and modulates biological behaviors, EMT and glycometabolism in bladder cancer. Data are presented as the mean ± SD of three independent experiments. *P < 0.05 and **P < 0.01 vs. the control group

To verify the relationship between TAZ and miR-942-3p in vivo, we subcutaneously injected cells with different expression patterns (negative control, TAZ knockdown or sh-TAZ with miR-942 overexpression) into nude mice. Tumor volume was measured every 5 days after injection. As shown in Fig. 8a-c, depletion of TAZ remarkably suppressed the tumor growth rate and tumor weight compared with control treatment. In addition, overexpression of miR-942-3p abrogated the inhibitory effects induced by TAZ knockdown. The miR-942-3p levels in tumor tissue in the three groups were confirmed by qRT-PCR (Fig. 8d). We next assessed the expression levels of TAZ, LATS2 and GAS1 in vivo. Immunochemical assays and western blotting indicated that TAZ knockdown upregulated the levels of LATS2 and GAS1 in tumor tissue and that miR-942-3p reversed these trends (Fig. 8e and f). These results confirm that miR-942-3p is vital for tumor growth mediated by TAZ in vivo. To illustrate our results more clearly, we created a schematic of the TAZ/miR-942-3p regulatory model (Fig. 8g).

## Discussion

TAZ is a key executor of the Hippo signaling pathway that regulates cellular proliferation, differentiation and tissue homeostasis [49, 50]. In recent decades, a great deal of evidence has indicated that dysregulation of TAZ contributes to cancer initiation and progression [51, 52]. The present study confirmed that TAZ was overexpressed in bladder cancer cell lines and tissue and related to cell survival, proliferation, migration and invasion.

The Hippo pathway has emerged as an important upstream signaling pathway in angiogenesis [53]. In detail, TAZ has been reported to be involved in VEGF-induced endothelial cell sprouting [54]. In addition, TAZ also enhances angiogenesis in EGFR wild-type non-small cell lung cancer cells [55]. According to these findings, we further evaluated the angiogenesis-inducing ability of conditioned medium acquired from control or TAZ-depleted cancer cells, and the results indicated TAZ has a key role in angiogenesis. However, the underlying molecular mechanism related to this interaction is worth investigating.

EMT is a transdifferentiation program in which epithelial cells acquire mesenchymal features. Interestingly, recent studies have indicated that EMT state regulates tumor migration, invasion, metastasis and resistance to clinical therapy. Moreover, EMT may lead to the emergence of cancer stem cells and trigger tumor initiation [16, 18, 21, 23, 56]. The Hippo signaling pathway has been reported to regulate the EMT process and mesenchymal characteristics [57–60]. In light of this, we examined EMT markers such as E-cadherin, Vimentin, N-cadherin, Fibronectin and Snail and found that TAZ knockdown reversed the EMT process. It is well known that numerous pathways and factors are involved in the modulation of EMT; therefore, further study is needed to validate the exact regulatory mechanism of the TAZ-induced EMT process.

TAZ and the Hippo pathway also participate in metabolic modulation, such as regulating glycolysis, lipogenesis, and glutaminolysis [10–14]. Interestingly, a growing body of evidence has verified that glycometabolism may modulate cell growth, migration and progression in bladder cancer [61–63]. While normal cells acquire energy for physiological processes from the oxidation of pyruvate, cancer cells rely on aerobic glycolysis to generate energy and compounds to support their aberrant growth and metastasis. This metabolic characteristic of cancer cells is termed the Warburg effect. The Warburg effect not only ensures an adequate supply of energy and nutrients but also provides an acidic microenvironment that enhances migration and invasion [64]. Therefore, clarification of the underlying mechanism of glycolysis in bladder cancer for clinical intervention and treatment is a meaningful pursuit. Our current results suggest that TAZ is vital for glycolysis in bladder cancer cells and functions by regulating the expression of PFKFB3, HK2 and GLUT1, which serve as key components in glycolysis. However, the deeper interactions between glycolysis and other biological effects mediated by TAZ are worth exploring and verifying in future studies.

Of note, redox homeostasis can be disrupted by TAZ inhibition-induced metabolic reprogramming in NF2-mutant tumor cells [14]. In the current study, apart from the suppression of glycolysis, ROS levels were remarkably elevated after TAZ knockdown in bladder cancer cells. A previous study reported that ROS stress could trigger a DNA damage response mediated by p53, inhibiting the proliferation and growth of tumor cells [65]. Therefore, it is reasonable to deduce that the elevated ROS levels may play a crucial role in the TAZ-induced alterations in biological behaviors. In future studies, we will investigate ROS stress-related signaling pathways and the interaction between ROS and biological functions in TAZ-depleted bladder cancer cells.

The TEAD protein family consists of four paralogous factors that function as nuclear DNA-binding proteins to modulate the transcriptional activity of downstream genes in response to the Hippo signaling pathway. Of note, the modulatory role of TEAD proteins depends on binding with TAZ or YAP in the nucleus, whose nuclear import is mediated by LATS1/2 [30, 31, 47]. MiRNAs participate in numerous biological processes, including tumor initiation, progression and metastasis [27, 34–36, 66]. Nevertheless, the mechanism underlying the dysregulated miRNAs in bladder cancer remains unclear. Therefore, we speculated that TAZ may exert its biological functions by enhancing the expression of miRNAs and, following miRNA sequencing, qRT-PCR, ChIP and luciferase reporter assays, illustrated that miR-942-3p is regulated by TAZ-TEADs in bladder cancer cells.

MiR-942 has been confirmed to play key roles in tumorigenesis and angiogenesis [45, 46, 67]. Our results showed that miR-942-3p was abundantly expressed in bladder cancer cell lines and tissue and served as a tumor promoter. Moreover, GAS1 and LATS2 were verified as direct downstream targets of miR-942-3p. LATS2 suppresses the transcriptional activation of Hippo pathway-related genes by phosphorylating TAZ [9, 28, 47]. GAS1 acts as a novel biomarker and inhibits proliferation, angiogenesis, EMT and glycolysis in human cancers [43, 44]. Based on previous studies and these results, our further experiments identified a positive feedback loop between TAZ and miR-942-3p that affects the biological impacts of GAS1.

## Conclusion

In summary, the present study verified a novel positive feedback loop between TAZ and miR-942-3p that regulates GAS1 expression and modulates biological behaviors, EMT, glycolysis and intracellular ROS levels in bladder cancer. TAZ, miR-942-3p and GAS1 may be potential therapeutic targets that can be exploited in clinical interventions for bladder cancer.

## Supplementary Information

**Additional file 1.**

**Additional file 2.**

## Data Availability

The datasets used and/or analyzed in this article were included within the article and the additional files. Please contact the corresponding author for data requests.

## Abbreviations

TAZ: Transcriptional co-activator with PDZ-binding motif
GAS1: Growth arrest-specific 1
EMT: Epithelial-mesenchymal transition
ROS: Reactive oxygen species
miRNA: Micro RNA
sh-RNA: Small hairpin RNA
siRNA: Small interfering RNA
3′-UTR: 3′- untranslated region
qRT-PCR: Quantitative real-time PCR
FBS: Fetal bovine serum
H&E: Hematoxylin and eosin
CCK-8: Cell Counting Kit-8
IHC: Immunohistochemistry
