# Supplementary material for: A positive feedback loop between TAZ and miR-942-3p modulates proliferation, angiogenesis, epithelial-mesenchymal transition process, glycometabolism and ROS homeostasis in human bladder cancer
Source: J Exp Clin Cancer Res. 2021 Jan 26;40:44. doi: 10.1186/s13046-021-01846-5 (PMC7836562; doi:10.1186/s13046-021-01846-5)
Supplement: Supplementary file 1 — Additional file 1. [file 13046_2021_1846_MOESM1_ESM.docx]

**A positive feedback loop between TAZ and miR-942-3p modulates proliferation, angiogenesis, epithelial-mesenchymal transition process, glycometabolism and ROS homeostasis in human bladder cancer**

Feifan Wang^1^, Mengjing Fan^2^, Yanlan Yu^3^, Yueshu Cai^1^, Xuejian Zhou^1^, Hongshen Wu^1^, Yan Zhang^1^, Jiaxin Liu^1^, Shihan Huang^1^, Ning He^1^, Zhenghui Hu^1^, Guoqing Ding^3*^, Xiaodong Jin^1*^

^1^Department of Urology, The First Affiliated Hospital, Zhejiang University School of Medicine, Hangzhou, Zhejiang, 310003, R.P. China;

^2^ Department of Pathology, Sir Run Run Shaw Hospital, Zhejiang University School of Medicine, Hangzhou, Zhejiang, 310016, R.P. China.

^3^ Department of Urology, Sir Run Run Shaw Hospital, Zhejiang University School of Medicine, Hangzhou, Zhejiang, 310016, R.P. China.

**Correspondence:**

Xiaodong Jin, Department of Urology, The First Affiliated Hospital, Zhejiang University School of Medicine, Hangzhou, Zhejiang, 310003, R.P. China;

E-mail: xiaodong-jin@zju.edu.cn

**Table S1. Detailed information of our own 20 bladder cancer patients is listed (cohort 1)**

| Patient number | Age at surgery | Gender | Grade | T | N | M | AJCC clinical stage |
| --- | --- | --- | --- | --- | --- | --- | --- |
| 1 | 53 | Male | High | T4b | N2 | M0 | 4 |
| 2 | 67 | Male | High | T4a | N1 | M0 | 4 |
| 3 | 40 | Male | High | T2b | N3 | M0 | 4 |
| 4 | 52 | Male | Low | Tis | N0 | M0 | Ois |
| 5 | 58 | Female | Low | Tis | N0 | M0 | Ois |
| 6 | 59 | Female | High | T1 | N0 | M0 | 1 |
| 7 | 78 | Male | Low | Tis | N0 | M0 | Ois |
| 8 | 81 | Male | High | T1 | N0 | M0 | 1 |
| 9 | 68 | Female | Low | Tis | N0 | M0 | Ois |
| 10 | 66 | Male | High | T1 | N0 | M0 | 1 |
| 11 | 68 | Male | High | T2a | N0 | M0 | 2 |
| 12 | 73 | Male | High | T4b | N1 | M0 | 4 |
| 13 | 61 | Female | Low | Tis | N0 | M0 | Ois |
| 14 | 62 | Female | High | T2 | N0 | M0 | 2 |
| 15 | 68 | Male | High | T1 | N0 | M0 | 1 |
| 16 | 84 | Female | High | T2a | N0 | M0 | 2 |
| 17 | 88 | Male | High | Tis | N0 | M0 | Ois |
| 18 | 86 | Male | High | T2 | N0 | M0 | 2 |
| 19 | 84 | Male | High | T2 | N0 | M0 | 2 |
| 20 | 48 | Male | High | T2b | N0 | M0 | 2 |

**Table S2.** **Detailed information of 30 bladder cancer cases of cohort 2**

| Patient number | Age at surgery | Gender | Grade | T | N | M | AJCC clinical stage |
| --- | --- | --- | --- | --- | --- | --- | --- |
| 1 | 63 | Male | Low | Ta | N0 | M0 | Ta |
| 2 | 79 | Male | High | Ta | N0 | M0 | Ta |
| 3 | 75 | Male | Low | Ta | N0 | M0 | Ta |
| 4 | 69 | Male | High | Ta | N0 | M0 | Ta |
| 5 | 48 | Female | High | T1 | N0 | M0 | 1 |
| 6 | 70 | Male | High | T1 | N0 | M0 | 1 |
| 7 | 54 | Male | High | T1 | N0 | M0 | 1 |
| 8 | 74 | Male | High | T1 | N0 | M0 | 1 |
| 9 | 72 | Male | High | T2 | N0 | M0 | 2 |
| 10 | 61 | Female | High | T2 | N0 | M0 | 2 |
| 11 | 77 | Male | High | T2 | N0 | M0 | 2 |
| 12 | 61 | Female | High | T3 | N0 | M0 | 3 |
| 13 | 51 | Male | Low | T3 | N0 | M0 | 3 |
| 14 | 65 | Female | High | T3 | N0 | M0 | 3 |
| 15 | 61 | Female | High | T3 | N0 | M0 | 3 |
| 16 | 46 | Female | High | T3 | N0 | M0 | 3 |
| 17 | 63 | Male | High | T3 | N0 | M0 | 3 |
| 18 | 65 | Male | High | T3 | N0 | M0 | 3 |
| 19 | 69 | Male | High | T3 | N0 | M0 | 3 |
| 20 | 79 | Male | High | T3 | N0 | M0 | 3 |
| 21 | 80 | Male | High | T3 | N0 | M0 | 3 |
| 22 | 62 | Female | High | T3 | N0 | M0 | 3 |
| 23 | 60 | Male | High | T3 | N0 | M0 | 3 |
| 24 | 73 | Male | High | T3 | N0 | M0 | 3 |
| 25 | 59 | Male | High | T3 | N0 | M0 | 3 |
| 26 | 73 | Male | High | T3 | N0 | M0 | 3 |
| 27 | 61 | Male | High | T4a | N0 | M0 | 3 |
| 28 | 64 | Female | High | T3 | N1 | M0 | 4 |
| 29 | 63 | Female | Low | T3 | N1 | M0 | 4 |
| 30 | 70 | Male | High | T2 | N2 | M0 | 4 |
